# Condition-Dependent Noise Correlations without Condition-Dependent Spike Counts

**DOI:** 10.64898/2026.05.08.723078

**Published:** 2026-06-12

**Authors:** Doyeon Kim, Matthew F. Panichello, Tirin Moore

## Abstract

The ability of the brain to encode information and control behavior depends on the coordinated activity of large and distributed neuronal populations. Correlations in neuronal spiking activity across trials of the same condition, or noise correlations (NCs), have been interpreted as a reflection of shared synaptic connectivity and as a contributing factor to the information capacity of neuronal populations. The impact of NCs on coding is most often considered in populations of neurons exhibiting robust condition-dependent information in their spike counts (SCs). However, theoretical work suggests that NCs could provide a source of condition-dependent information separate from SCs. We examined the activity of large neuronal populations in prefrontal cortex of macaques while they performed a spatial delayed response task composed of visual, memory, and motor epochs. We found that pairs of neurons that displayed visual, memory, and motor selectivity in their SCs often exhibited selectivity in their NCs, independent of spike count. However, we also found that pairs of neurons without SC selectivity during the different task epochs nonetheless exhibited condition-dependent NCs. Moreover, we found that the magnitude of condition-dependent NCs were largely comparable across neuronal pairs with or without SC selectivity. These results demonstrate that correlated variability in spiking activity can be condition-dependent even in the absence of condition-dependent SCs.

## Full Text Availability

The license terms selected by the author(s) for this preprint version do not permit archiving in PMC. The full text is available from the preprint server.

